# Case Report: Coronary vasospasm precipitating STEMI and polymorphic ventricular tachycardia—a case of cardiotoxicity from 5-FU based chemotherapy in a 41-year-old woman

**DOI:** 10.3389/fcvm.2025.1594338

**Published:** 2025-09-09

**Authors:** Ammar Hasnie, Zachary Neace, Kristin Ellison, Aaron Hesselson, Karam Ayoub, Mohammed Al-Yafi, Amit Arbune

**Affiliations:** Department of Cardio-Oncology, Gill Heart & Vascular Institute, University of Kentucky, Lexington, KY, United States

**Keywords:** FOLFOX, 5-FU, cardiotoxicity, ventricular arrhythmia, MINOCA

## Abstract

**Background:**

Intravenous 5-fluorouracil (5-FU), in combination with oxaliplatin and folinic acid, makes up a chemotherapy regimen commonly referred to as FOLFOX. It is well-established for its efficacy in patients with advanced colorectal cancer; however, the benefits are accompanied by potential side effects that warrant careful consideration. Common toxicities can range from nausea and vomiting to neuropathy. Cardiotoxicities related to FOLFOX and fluoropyrimidines in general are extremely rare but can easily be fatal.

**Case presentation:**

A 41-year-old woman who was recently diagnosed with stage IIIc colorectal cancer after she underwent a subtotal colectomy was admitted to the Oncology clinic for further treatment. Given her excellent performance status and lack of comorbidities, she was started on adjuvant FOLFOX therapy. Three days after her first dose, she presented to the emergency room with several episodes of self-limited substernal chest pain. An electrocardiogram was performed, which showed ST-segment elevation concerning for acute myocardial infarction. She was taken emergently for a coronary angiogram, which revealed no evidence of obstructive coronary artery disease or spontaneous coronary artery dissection. Her presentation was most consistent with coronary vasospasm secondary to her recently started chemotherapy regimen. She was monitored in the cardiac critical care unit; the next day, she developed a breakthrough chest pain and subsequently developed polymorphic ventricular tachycardia with loss of consciousness. It was found that she had suffered a breakthrough coronary vasospasm precipitating a life-threatening arrhythmia. She was started on calcium channel blockers and nitrates with the aim of preventing further episodes of her hypersensitivity-induced vasospasm, and she was eventually successfully rechallenged with a Nordic FLOX bolus-based regimen.

**Conclusions:**

In an unusual fashion, our patient developed ST-segment elevation myocardial infarction (STEMI) caused by coronary vasospasm, followed by delayed polymorphic ventricular tachycardia approximately 24 h later. This dual-phase presentation and subsequent successful rechallenge with bolus-based 5-FU chemotherapy have not been previously reported.

## Introduction

FOLFOX is a synergistic combination of three potent chemotherapy drugs: 5-fluorouracil (5-FU), oxaliplatin, and leucovorin. This regimen has solidified its status as a standard of care in cancer treatment, particularly for colorectal cancer and other gastrointestinal malignancies (1). Oxaliplatin exerts its cytotoxic effects through platinum analogs binding DNA and forming interstrand cross-links, leading to the inhibition of DNA synthesis and function (2, 3). 5-FU also augments the disruption of DNA synthesis through the inhibition of thymidylate synthase (4). Combination therapy, in the form of FOLFOX, has been shown to improve response rates, reduce the risk of cancer recurrence, and increase overall survival rates as adjuvant therapy in patients with advanced colorectal cancer (1, 5, 6). Despite its efficacy, rare cardiotoxicities can prove fatal, underscoring the need for vigilant monitoring to balance benefits and risks in patients undergoing this chemotherapy regimen.

## Case presentation

A 41-year-old Caucasian woman with a past medical history of hypertension was diagnosed with stage IIIc left-sided colon adenocarcinoma after she presented with a large bowel obstruction secondary to her tumor burden. She underwent a subtotal colectomy, and a postoperative pathologic assessment of her surgical margins was noted to be clear. She was started on adjuvant FOLFOX therapy with curative intent (5-FU 400 mg/m^2^ bolus followed by 2,400 mg/m^2^ infusion, oxaliplatin 85 mg/m^2^, and leucovorin 400 mg/m^2^). She was planned to undergo an infusion every 28 days for a 6-month course. Approximately 72 h after her first dose, she presented to the emergency department with complaints of several episodes of self-limiting substernal chest pain that radiated down her left arm. At the time of arrival to the emergency department, she was free from chest pain. On presentation, she was afebrile with a blood pressure of 110/69 and a pulse rate of 102 beats per minute. She was on room air and her oxygen saturation rate was 97%. Her physical examination was benign, except for tachycardia, her heart had a regular rhythm without murmurs, her lungs were clear to auscultation bilaterally, and there was no lower-extremity edema.

**Figure 1:**
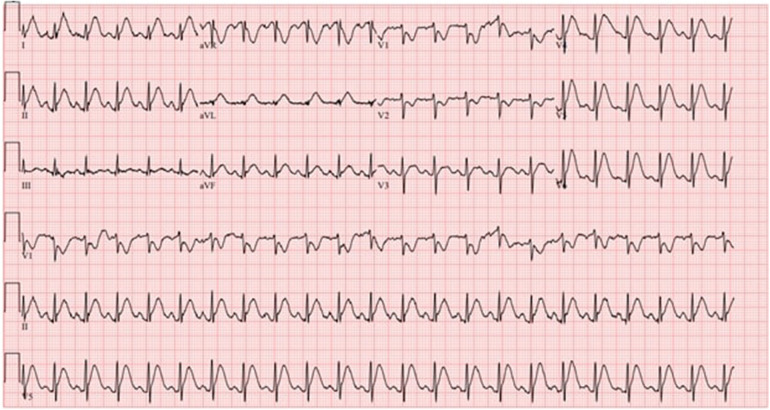
ECG shows sinus tachycardia with ST elevation concerning for inferolateral injury or acute infarct with right ventricular involvement.

Her initial electrocardiogram (ECG) showed a normal sinus rhythm, with a heart rate of 100 beats per minute, without any ST or T wave changes concerning for ischemia. However, while waiting in the emergency room, she again developed substernal chest pain and a repeat ECG was done. This time it showed ST elevations in the inferior and lateral leads concerning for acute coronary syndrome (ACS, Figure 1). High-sensitivity troponin level was 55 ng/L (upper level of normal 14 ng/L) and the repeat level was 49 ng/L. Her B-type natriuretic peptide (BNP) was 321 pg/mL (normal range < 449 pg/mL). The results of her thyroid tests were normal. A transthoracic echocardiogram (TTE) showed that her left ventricular ejection fraction (LVEF) was low normal at 50%–55% with no regional wall motion abnormalities. Given her chest pain and acute ECG changes, she was emergently taken for a coronary angiogram, which revealed angiographically normal coronary arteries. She was diagnosed with transient coronary vasospasm associated with 5-FU chemotherapy, which was the cause of her chest pain and acute ECG changes. She was admitted to the coronary care unit (CCU) for overnight observation. She was also started on an intravenous (IV) nitroglycerin drip as prophylaxis for preventing further episodes of coronary vasospasm.

**Figure 2:**
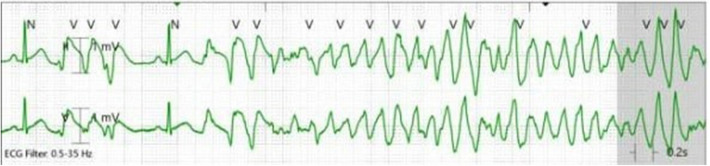
Telemetry strip showing the development of polymorphic ventricular tachycardia.

In the CCU, she remained free from chest pain and without any arrhythmias noted on telemetry. Her IV nitroglycerin drip was continued for 14 h at 0.25 μg/kg/min. Because of borderline hypotension (as low as 86/49 mmHg), the initiation of oral nitrate therapy was delayed. Approximately four hours after the IV nitroglycerin drip was stopped and before she received an oral long-acting nitrate, she experienced a breakthrough chest pain. A few seconds later, she experienced a loss of consciousness, and telemetry revealed polymorphic ventricular tachycardia (PMVT) (Figure 2). Chest compressions were started, and by the time defibrillator pads were placed on her, she spontaneously converted to a normal sinus rhythm. An ECG obtained shortly after the event revealed a normal sinus rhythm without any ST or T wave changes to suggest acute ischemia.

Given her episode of a malignant arrhythmia with loss of consciousness, electrophysiology, medical oncology, and cardio-oncology were consulted for further recommendations. As her coronary angiogram was unremarkable and the events were temporally related to her first dose of FOLFOX chemotherapy, a diagnosis of coronary vasospasm was solidified. However, her episode of breakthrough ischemia and PMVT became concerning for possible future out-of-hospital events. In the setting that this was a provoked event secondary to coronary vasospasm from 5-FU, she was medically managed with calcium channel blockers (CCBs) and nitrates. A cardiac MRI (CMR) showed a normal-sized left ventricle with a mildly reduced global systolic function (LVEF 48%). There were no regional wall motion abnormalities or evidence of scar on late gadolinium enhancement sequences. The CMR showed no evidence of myocarditis or inflammatory processes with normal T1 and T2 mapping sequences, further confirming no alternative etiology for PMVT. She was monitored in an ICU setting for 72 h with no further episodes of arrhythmias or chest pain. She was agreeable to getting discharged with a LifeVest Wearable Cardiac Defibrillator (Zoll Medical, Chelmsford, MA, USA). Prior to being discharged, she had a comprehensive discussion with both medical oncology and cardio-oncology about her treatment plan. Unfortunately, FOLFOX therapy was found to be superior to alternative forms of chemotherapy that could be offered. With the collaboration of medical oncology and cardio-oncology, a decision was made to rechallenge her with Nordic FLOX bolus dosing in an inpatient setting with maximal medical therapy.

The patient was rechallenged with the institute protocol using 5-FU bolus chemotherapy and pretreated with long-acting nitrates and calcium channel blockers 30–60 min prior to her chemotherapy infusion in the inpatient setting. The detailed chemotherapy regimen included fluorouracil, leucovorin, and oxaliplatin (oxaliplatin 85 mg/m^2^ given as a 2-h infusion followed by a bolus injection of 5-FU 500 mg/m^2^ and 30 min later a bolus injection of leucovorin 60 mg/m^2^). The patient completed all 12 planned cycles of Nordic FLOX chemotherapy, with the initial four rounds as an inpatient and the subsequent infusions as an outpatient without any recurrence of cardiac events. On follow-up imaging and surveillance, she has shown no evidence of disease progression or cardiovascular complications. Her follow-up echocardiogram showed recovery of her LV systolic function with an LVEF of 59% and global longitudinal strain >−18%. She was taken off the LifeVest Wearable Cardiac Defibrillator after the completion of the chemotherapy treatment. She also wore a patch monitor for 14 days, which did not show any ectopy or evidence of arrhythmias, either atrial or ventricular.

## Discussion

While FOLFOX therapy has become standardized as the gold standard for advanced colorectal cancer, the synergistic combination of three potent chemotherapy drugs also possesses a broad spectrum of side effects (7). The reported rates of incidence of cardiotoxicities from FOLFOX range from 1% to 10% depending on the schedule, dosing, and route of administration (7, 8). There are multiple proposed mechanisms for 5-FU cardiotoxicity; however, coronary vasospasm is most commonly thought to be directly related to 5-FU (9, 10). 5-FU exerts its effects primarily during the S-phase of the cell cycle. Its active metabolite, 5-flurodeoxuridine monophosphate (5-FdUMP), functions by inhibiting thymidylate synthase, a crucial enzyme in DNA synthesis. This inhibition disrupts DNA production, causing an imbalance in cell growth and eventually leading to cell death (4). 5-FU and its active metabolites can impair the normal functioning endothelium, leading to an elevated release of endothelin-1, a vasoconstrictor, and a diminished release of prostacyclin, a vasodilator causing coronary vasospasm (11–14). Alternative theories of 5-FU-induced coronary vasospasm also include direct toxicity to smooth muscle cells of the coronary arteries, causing arterial vasoconstriction or an inflammatory response within the arterial walls, leading to increased production of inflammatory mediators promoting vasoconstriction and subsequent coronary vasospasm (12–14). Cardiotoxicity has also been reported with capecitabine, the oral prodrug of 5-FU, further supporting a class effect among fluoropyrimidines.

Cardiotoxicities can range from subclinical symptoms manifesting as ECG changes to sudden cardiac arrest (11, 15). Notably, recent studies have found that cardiac-related adverse events were more commonly seen in younger patients without any underlying coronary artery disease, such as our patient (16). The reason for this is probably multifactorial, but a potential etiology can be related to specific gene variations that affect vascular function and that can increase the susceptibility to coronary vasospasm induced by 5-FU (15, 16). Clinically, patients with cardiac events tend to present after the first cycle of chemotherapy, and such events are less likely to occur with subsequent infusions (17). Given our patient's temporal association of her cardiac events with her first infusion of FOLFOX, and with other causes being ruled out, she was diagnosed with 5-FU-associated coronary vasospasm. After her coronary angiogram, she was appropriately started on vasodilator therapy with an IV nitroglycerin drip and then transitioned to a long-acting nitrate. Despite this, she had breakthrough coronary vasospasm precipitating polymorphic ventricular tachycardia. Given her event with nitrate monotherapy, she was also started on a non-dihydropyridine CCB. A large study recently found that among this patient population with 5-FU coronary vasospasm, rechallenge after pretreatment with CCBs and/or nitrates in collaboration cardio-oncology was safe and allowed continued 5-FU therapy (18). Unfortunately, alternative therapies are inferior to FOLFOX for patients with advanced colorectal cancer (19). Deciding to end treatment prematurely could lead to inadequate cancer management and negatively affect overall survival rates. Emerging evidence suggests that 5-FU cardiotoxicity risk may depend on the method of administration, with continuous infusion linked to high coronary vasospasm compared with bolus dosing (20). In our patient, rechallenge with the bolus-based Nordic FLOX regimen with prophylactic antispasmodic therapy was successful, indicating that modifying the infusion strategy along with vasodilators may mitigate the risk of toxicity in select patients (21, 22).

Our case reaffirms the known association of cardiac adverse events related to FOLFOX therapy. In an unusual fashion, our patient presented with dual complications, for example, her initial presentation as a ST-segment elevation myocardial infarction (STEMI) secondary to coronary vasospasm that was subsequently followed by a delayed development of PMVT approximately 24 h after her initial presentation. To our knowledge, there have been no other reported cases of a patient developing both an initial and delayed complication following the initiation of 5-FU-based chemotherapy where such patients were successfully rechallenged and 5-FU-based chemotherapy was completed. Recognition of this diagnosis is time-sensitive, given the chances of high mortality associated with patients presenting with myocardial infarction or ventricular arrhythmias related to unopposed coronary vasospasm.

## Data Availability

The original contributions presented in the study are included in the article/Supplementary Material, and further inquiries can be directed to the corresponding author.
